# Editorial: In Honour of Professor Margaret Brazier: Memories of Margot

**DOI:** 10.1093/medlaw/fwaf022

**Published:** 2025-09-12

**Authors:** Sara Fovargue, José Miola, Beverley Clough, Rob Heywood, Sarah Devaney, Alexandra Mullock, Elizabeth Chloe Romanis

**Affiliations:** School of Law, University of Sheffield, Sheffield, England; Leicester Law School, University of Leicester, Leicester, England; Manchester Law School, Manchester Metropolitan University, Manchester, England; UEA Law School, University of East Anglia, Norwich, England; Department of Law, University of Manchester, Manchester, England; Department of Law, University of Manchester, Manchester, England; Durham Law School, Durham University, Durham, England

## I. INTRODUCTION

Professor Margaret ‘Margot’ Brazier died on 4 March 2025. Margot was a former Editor-in-Chief of this journal, and a key figure in the life of the current Editorial team, members of our Editorial Advisory Board, and in the lives of many of those previously involved with the journal. We wanted to take this opportunity to pay our respects to Margot and reflect on and recognise her significant role in the field as a whole, as well as to us personally.

We have written a joint editorial with three members of the Editorial Team of *Medical Law International*, many of whom also benefited from Margot’s wisdom and tutelage. The same editorial will be published in both journals, as a small way to honour one of the central tenets of Margot’s professional philosophy—collaboration.

Alongside this, we have asked some of Margot’s peers for their professional and personal reflections on her work and their experiences with her. We are enormously grateful to all involved for their willingness to take the time to write, especially those who are no longer working in the field.

We hope that some of what follows highlights the esteem in which Margot was held, how much she means to us, and the loss we all feel. Our thoughts are with Rodney, her husband, and Vicky, her daughter.

## II. SARA FOVARGUE, PROFESSOR OF LAW, UNIVERSITY OF SHEFFIELD

In 1996, I was struggling with my PhD studies and was interviewed for a research assistant position at the newly created Institute of Medicine Law and Bioethics (IMLAB), a research collaboration between the universities of Manchester and Liverpool. It was, I think, a four-person panel, including Margot and John Harris. It was Margot who stood out to me. Having done my homework, I knew who she was—the chair of the Animal Procedures Committee. As a committed vegetarian and writing a PhD on xenotransplantation, Margot was clearly my ‘enemy’. How wrong I was.

I was appointed as a research assistant based at Liverpool. While grateful for the opportunity and I learnt a lot, as time went by it was clear that I was not happy—and Margot saw that. She started to invite me to events in Manchester—professional and social. She asked me to read and comment on draft articles (‘What? How could I possibly have anything to say about her work?’), talked to me about restarting my PhD (which I’d paused), encouraged me to write, and became my academic mentor (depending on our mood she could be like my mum, aunt, sister, friend, or all in one).

In 1998, I was fortunate to be appointed as Margot’s research fellow towards the end of an European Commission funded project on reproductive choice and the control of fertility. Margot pushed me to start presenting at conferences (to conquer my fear of public speaking) and then supported me in my search for my first academic position. From then on, Margot was an integral part of my life, and it is hard for me to express the impact that she has had on me—professionally and personally. Above all, Margot was my friend, and I am unbelievably lucky to have shared so much with her.

Margot’s intellect cannot be matched. I have, though, tried to follow and model other aspects of her professional conduct and behaviour. I learnt from Margot the importance of supporting and encouraging colleagues, especially those starting out, and of the joys of writing, researching, and teaching with others. Margot taught me that it is ok to write and work alone, but that it is definitely more fun (and challenging) to work with others. I observed her sharing opportunities that came her way; opportunities that she could have just kept for herself, but rarely did. I have tried to mirror this too. Her professional courtesy was impeccable—always acknowledging colleagues with whom she worked and developed ideas with, and those who had commented on drafts of her work.

Margot was kind, funny, generous, and with an astonishing knowledge of many subjects and topics. As I drove away from having had a cup of tea and a natter at Margot’s, I was always left a little stunned by how little I knew about so very much. And yet she never made me feel like that—Margot listened carefully, spoke wisely, and is very much missed.

## III. JOSÉ MIOLA, PROFESSOR OF LAW, UNIVERSITY OF LEICESTER

I had the pleasure, honour, and privilege to be one of Margot’s PhD students, and I have a few stories to tell about that experience that speak to Margot’s character and work as an academic. The first is the first time I met her. I had applied for a PhD scholarship at Manchester, and I had been offered an interview with Margot. She immediately put me at ease, and we spent a good half hour of her very precious time talking in depth about a research proposal that she had clearly read and thought about. But what really stood out was that, at the end of the interview, she presented me with copious notes that she had made—in her characteristic, virtually unreadable handwriting—about how my research proposal could be improved. She explained that, whether I came to Manchester or decided to do the PhD elsewhere, my current proposal was problematic, but with the tweaks that she had written down, it would be workable. She had devoted time and mental energy to help someone whom she had never met and was not even a student (let alone a student of hers). The benefit of her expertise to me was incalculable, but of course, there was no benefit to her. As I got to know her, it became clear that not only was I not the only recipient of this generosity of time and effort on her part, but that this is something that she always did for anyone.

I could also talk about her office—a palace of papers that covered her desk, chairs, and even her computer. I more than once spent at least 15 minutes of a supervision meeting helping her to look for my work in her office among the piles of papers. When found, it would always be covered in that scrawling handwriting and packed with insight.

But her lasting legacy will be us—medical lawyers (and ethicists) like those of us writing this who have worked with her and been helped by her. She fostered a strong sense of community between us and made sure that we always knew that she would be there for us. She said to me once that PhD students were for life, not just for three years. When the news of her passing broke, our community showed its togetherness. Messages were flying around with people offering support to each other and checking on everyone’s welfare. Her kindness towards others had been passed on to us, and that too is something to cherish.

## IV. BEV CLOUGH, PROFESSOR OF LAW AND SOCIAL JUSTICE, MANCHESTER METROPOLITAN UNIVERSITY

As a third-year undergraduate, I was starting to question why I had chosen to study law. This changed, however, when I took the Principles of Law, Medicine and Ethics course at the University of Manchester. I vividly remember sitting in the lecture theatre and listening to Margot, rapt by the introduction to medical law and the historical and societal context for the course. I was hooked. Margot’s ability to hold the interest of the students and to recount the stories behind the cases was remarkable. It was this emphasis on the human side of medical law and the stories and social contexts underpinning the judgments and policy decisions we were studying that struck me, and which has shaped my own research and teaching. Later in the year, I was incredibly nervous when I went to speak to Margot in her office, sitting at the table surrounded by the iconic stacks of paper. I need not have been nervous; Margot was kind, open, and generous. I mentioned that I had undertaken work experience in a clinical negligence department in Manchester, yet I was feeling unsure about a career as a solicitor. The encouragement by Margot to explore further academic study and to look into the MA in Health Care Ethics and Law was hugely significant in opening up a pathway that had never been in consideration.

As a postgraduate student in the wonderful Centre for Social Ethics and Policy (CSEP), I quickly began to feel part of a community. The generous and supportive culture that Margot led was felt even as an MA student. The regular CSEP Senior Seminar Series was a highlight, witnessing individuals whose books and articles we read on the course in (sometimes fierce, but always constructive) debate was both daunting and inspiring. It was as an MA student that I worked with a fellow student on the indexing for the 5th Edition of *Medicine, Patients and the Law*. Not only did this open my eyes to the process of publishing, it also enabled me to work closely with Margot. I was struck again by how open and generous she was, and how she treated me and the other student as equal and valued colleagues too.

Later, as a PhD student and Graduate Teaching Assistant, Margot became both a colleague on teaching teams and a collaborator on one of my first academic papers. I feel privileged to have benefited from Margot’s wisdom and care during this time, giving me confidence when I had wobbles, but, perhaps more importantly, modelling a way of being an academic that I know now, with hindsight, is incredibly rare. I gained a clear sense of how important a collegiate and supportive academic culture is and how fragile this can be. When taking my first steps as a new lecturer, feeling overwhelmed and woefully underprepared, I tried to take inspiration from Margot—both her approach to lecturing and emphasising the human stories underneath the cases, but also in how I always make time to offer support to students and colleagues.

## V. ROB HEYWOOD, PROFESSOR OF MEDICAL LAW, UNIVERSITY OF EAST ANGLIA

It is difficult to express in words the respect and admiration I have for Margot. As a professional, she was intelligent, articulate, driven, kind, and supportive. As a friend, she was interested, caring, a great listener, witty, and fabulous company. I did not know Margot as well as some of my colleagues on the Editorial Team, but she was still a tremendous source of inspiration for my research and helped me immensely throughout my academic career. To say that she influenced the way in which I think about the discipline of medical law is an understatement.

After reading her insightful articles on consent, which helped to shape my thesis as a PGR student, I first met Margot in person at a conference that was held at the University of Manchester. As I was just starting out in my career at that time, I remember feeling extremely nervous when attending this event surrounded by so many established academics. Margot made such an effort to come and speak to me and showed a genuine interest in my work. If I arrived feeling nervous, I left that event feeling ten feet tall, and that was because of Margot’s thoughtfulness. She did not have to make that effort to speak to me as an unknown PhD student, and other distinguished Professors might not have done, but that was a testament to her character and her ethos of supporting junior colleagues coming through the ranks. I am sure I am not the only one with a story like that.

I first read Margot’s brilliant textbook—*Medicine, Patients and the Law—*as an undergraduate student at Sheffield Hallam University. I recall being so impressed by the breadth and depth of her analysis and the way in which she challenged readers and questioned received wisdom. The main beauty of the book, however, was its accessibility and ability to engage readers from different backgrounds. After working with healthcare professionals in Sheffield, I soon began to realise that it was not only a book for lawyers, but also a text which clinicians were equally familiar with. I have lost count of the number of times I have referred to the book as an undergraduate student, a PGR student, and a teacher.

When I first encountered *Medicine, Patients and the Law*, never in my wildest dreams would I have imagined that one day I would be invited to co-author the book, and when Margot and Emma Cave approached me to collaborate on the 7th edition, I felt immensely proud.

Nevertheless, I was also a little anxious as I had never before embarked upon a project of such magnitude, and the weight of expectation felt heavy as I knew that it would be so important to maintain the high standard of scholarship that defined the previous editions. I was conscious of the heavy workload that such a writing task would entail too. As it happened, I did not need to worry about any of those things, as the encouragement, support, and assistance I received from both Margot and Emma were outstanding and made my first outing on the book a thoroughly enjoyable experience. I have fond memories of Margot advising me on the difficult and lengthy chapters on capacity and consent and providing me with lots of hints and tips as to what had worked well before, and the dangers to avoid. During the drafting of the chapter on criminal process and medical malpractice, which involved me having to get to grips with quite a lot of new material, Margot’s input was invaluable as she helped me to navigate this interesting area that she was so passionate about.

Alongside the benefit of her wisdom on the academic side of the book, I remember some of the other things she that did, things which may seem trivial at a glance but which were incredibly important to me as someone who had joined the team for the first time. For example, Margot went to special lengths to check that the acknowledgements to my wife Nicola and my daughter Isabella were correct and exactly as I wanted them to appear in the opening section of the book. Small gestures such as these count for so much, and Margot always paid careful attention to them. Another time, Margot, Emma, and I were laughing and joking about which colour to select for the front cover of the 7th edition. Somehow, I managed to persuade them that red would be a good idea to reflect the fact that a Manchester United fan had joined the team of authors. They were kind enough to indulge my request. That showed another side to Margot; throughout my time working with her, I always found her to have a lovely humour and subtle sense of mischief about her, which projected the warmth in her personality.

Perhaps the nicest thing I remember, though, is not the first time I got to see the final published version of the 7th edition of *Medicine, Patients and the Law* in hard copy. That was fantastic, but it is something that came shortly afterwards that sticks in my mind the most. I do not think that I had ever given Margot my home address, but a few weeks after the birth of Isabella, the most pleasant surprise arrived—a beautiful green jumper with a big bumblebee on the front for Isabella from Margot. Isabella knows that it is ‘Margot’s jumper’, and we will cherish it and keep it safe forever.

Margot was very special and she leaves behind a fabulous legacy. She will be remembered for all the right things and I will miss her dearly. She will always have a place in the history of medical law and will never be forgotten by anyone who has ever had the pleasure to meet and work with her.

## VI. SARAH DEVANEY, PROFESSOR OF HEALTHCARE LAW AND REGULATION, UNIVERSITY OF MANCHESTER

I first had the chance to speak with Margot when I was invited to interview for a post as a Graduate Teaching Assistant, which would offer teaching experience and pay a stipend to support me in my PhD studies. I was really fortunate that, despite coming up against the formidable Amel Alghrani as an alternative candidate, Margot, together with John Harris and Suzanne Ost, lobbied the Law School to provide two such posts to take both Amel and I on board. I know that Amel and I would agree that this move was one that changed our lives.

Having made the move to becoming a PhD candidate from a career in practice as a solicitor, I was used to an expectation of in-person presence in the office between Monday to Friday from 9 am to 5 pm at the very least. My contract at the University started on the 1 September 2004 and, on that day, I turned up at the Williamson Building on Oxford Road at 9 am. You can imagine my surprise to find that, apart from the wonderful stalwarts in the Professional Support office, the building was otherwise almost entirely deserted. To my immense relief, the exception to this was, of course, Margot, who cheerfully welcomed me, took me for lunch, and set me off on my research.

Margot took her responsibilities to teaching and research equally seriously and enjoyed both immensely. This balance of commitment also applied to her supervision of my combined teaching and research responsibilities. On the teaching front, she invited me to observe her first lecture of the term in her undergraduate medical course, then titled Law, Medicine and Ethics. Margot’s teaching style, as many thousands of former students will remember, combined enormous enthusiasm for the subject matter with a genuine curiosity about the views of those present in the session. I remember being both swept along with that enthusiasm, on that occasion, for the role that ethical analysis could play in helping lawyers consider knotty problems of life, death, and everything in between in the healthcare context, and feeling enormously daunted: how on earth to follow that?

Margot was disarmingly modest in all things and, when in the early days of my teaching career I confessed to finding that holding lectures and seminars instilled a combination of thrill and fear, swiftly followed by exhaustion, she cheerfully admitted to still seeing teaching as involving acting, playing the part of a confident and knowledgeable expert in the field, even though one was not quite such a person. Such reflections represented Margot’s generous instinct to support colleagues in both the obvious and perhaps more hidden challenges of an academic career.

I still have the piece of paper on which Margot wrote her peer review of my first session of seminar teaching on 13 October 2004. Once I had worked out how to decipher her infamous hieroglyphics, her encouragement shone through, even to the extent of providing a footnote to explain that a lower score on one of the elements was not that I wasn’t doing the thing (encouraging discussion) but that there was room for improvement. Margot’s wonderful propensity to see more in students and supervisees than they knew they had was a major contributor to their success.

As a PhD supervisor, Margot tolerated my utter cluelessness about the process with great discretion. Amel and I will never forget the moment when, after a number of months of our study, Margot had evidently decided that she really did need to intervene to offset our deepening confusion. She gathered us together for an unusual joint supervisory chat and explained to us both with enormous clarity the topics of our own PhDs and the differences between them. It was a welcome lesson in ensuring that one is clear about the scope and purpose of a piece of research and it helped keep us on the research tracks we have continued to engage with for many years. Margot was a great advocate for her supervisees, ambitious for us, encouraging and advocating for our involvement in research opportunities from the start.

One of my first acts as a GTA was to get pregnant with my first child. I was terrified at how this might affect my position in the department, but was swiftly reassured by Margot’s happiness at the prospect of the new baby. Many of my other CSEP and health law colleagues with whom Margot was working, and on whom she was relying for teaching or research support, had the same idea around that time, resulting in a notable flurry of babies arriving in short order. The effect on Margot’s working life must have been disruptive to put it mildly—finding interim replacements for critical roles in funded research, or cover for demanding teaching needs, must have been enormously challenging. No one could have blamed her had she questioned her professional bad luck on this front. Naturally, though, Margot was never anything other than delighted for colleagues and their growing families.

As a colleague, Margot was open about the challenges of life in academia but always with humour, to the extent that another colleague was once heard to wonder why laughter could always be heard coming from her office during CSEP meetings. The thought of her irresistible wit and sense of fun still brings a smile. Not many people outside the health law sector will know that within the UK there is an alternative currency to the Great British Pound. It takes the form of the KitKat and was established and deployed as an incentive by Margot with great regularity and devastating effect. Reluctant to answer a question in class? Would a KitKat help you to commit to a view on the appropriate parameters of assisted dying? Struck by writer’s block? How about a KitKat for writing 500 words by the end of the day? The remarkable thing about this currency was that the incentive to action was very effective, but I can’t remember an actual KitKat ever changing hands! Another example of Margot’s ability to discern what really makes people tick.

I can never go far wrong when I ask myself in work and in life, ‘what would Margot do?’ The answer is usually to help and support other colleagues, even those who one might think would not need it; take the opportunity to find humour and humanity in work challenges, be curious, make the most of time with friends. Margot, together with her CSEP colleagues and wider networks in healthcare law and ethics, established an environment of curiosity, drive, support, and passion for the area, which now, to my mind, pervades the discipline as a whole. Within that, Margot’s love, friendship, and support were remarkable to me, at first in its very existence, and then always in its depth and persistence. I owe her so much and will always be so grateful.

## VII. ALEX MULLOCK, SENIOR LECTURER IN MEDICAL LAW, UNIVERSITY OF MANCHESTER

My first conversation with Margot was in 2008 when I nervously spoke to her on the phone about a funded PhD opportunity. It was lunchtime and I was sitting in my car in the staff car park of a sixth form centre in Staffordshire where I taught A Level law. I was utterly in awe of ‘Professor Margaret Brazier’ after reading (and loving) her diverse work while studying medical law as an undergraduate and postgraduate, and so it was with great trepidation that I dialed her Manchester number. She answered the phone quickly and I immediately apologised for wasting her time, before asking her to confirm my expectation that there was no point in me applying for the PhD as I was clearly deluded to think I stood any chance of success. With great kindness, Margot dismissed my worries, asked about my career history, thanked me for calling, and encouraged me to apply straight away. This brief phone conversation in 2008 changed my life.

Getting to know Margot as the ‘Boss’ of the AHRC project on the impact of the criminal law on medical ethics and practice was wonderfully surprising. Besides her kindness—which I soon realised was a defining characteristic—and her propensity for seeing the best in people, Margot was so interested in others, especially their children and their dogs. She was also great fun to be around. Our chatty, humorous project team meetings were the highlight of the week. As a baffled PhD candidate embarking on yet another career path in my 30s, wondering what on earth I was meant to contribute, the project gifted me an exceptionally lucky opportunity to join Margot’s awesome team (consisting of Suzanne Ost, Andrew Sanders, Anne-Maree Farrell, Charles Erin, Amel Alghrani, Danielle Griffiths, Melinee Kazarian, and Kate Bradbury). It also offered a uniquely valuable introduction to an incredible group of researchers in the wider community of health law and ethics scholars, which Margot had helped to establish. Margot also supervised my PhD for the first year while my main supervisor, Suzanne Ost, was on maternity leave. For one of our first supervisions, my childcare arrangements had fallen through, and so my daughter, Amelia, had to come along. I was concerned that the surprise presence of a toddler in a supervision would not please Margot, but rather than being annoyed, she seemed delighted to meet Amelia and offered paper (from her table of paper chaos), plus pens to keep Amelia amused. Though Margot only supervised me formally for the first year, she continued to be a wonderful mentor throughout my PhD and far beyond.

Teaching a course with Margot was a great privilege, particularly observing how she brought clarity to complex issues and created thought-provoking examples that brought the topic alive for students, all aided by her quiet humour. It helped that this was delivered in her unexpectedly powerful voice that needed no amplification, even in larger lecture theatres. During these years, Margot and I had lots of wonderful conversations in my car when I gave her a lift home from work. Embarrassingly, I would regularly have ‘lost’ my car in the multi-storey car park and Margot would join me on the search as I tried to remember where I had parked that morning. We would laugh together as we searched each floor, and though she had every reason to think I was an idiot, she never made me feel like one.

So much about Margot besides her powerful voice belied her small stature. Her huge generosity and kindness, her enormous intellect, her immense wisdom, and her great humour made her an incredibly special mentor, colleague, and friend. When Margot became ill in the final chapter of her life, we also discovered how very brave she was. Margot was a truly extraordinary person. As Sarah Devaney has pointed out, when we are faced with a difficult choice, if we ask ourselves, ‘what would Margot do?’ it reliably points to the most compassionate and judicious option. I feel so grateful to have known and loved Margot.

## VIII. ELIZABETH CHLOE ROMANIS, ASSOCIATE PROFESSOR IN BIOLAW, DURHAM UNIVERSITY

I can’t remember the first meeting with Margot, only that I was absolutely terrified to say something stupid in front of her. I chose Manchester for my undergraduate studies in 2012 because I knew I liked the idea of medical law. When hunting around for different universities, I came across lots of her work. She was at the forefront of why Manchester was the place that it is. Then I had arrived at Manchester, read more of her writing (in awe of how she could be an expert on quite so many things) and seen her command attention in a lecture theatre in a way I had never seen before (or much since). It was easy to be intimidated as a student by someone with so much knowledge and presence. It did not take long though to realise that Margot, despite how brilliant she was, was the last person to be afraid of. Rather, she was the sort of person whose brilliance was only exemplified by her kindness and interest in others. Margot was always genuinely interested in what everyone had to say and was in no way hierarchical about it.

I will always be grateful to Margot for how generous she was with her time. My favourite memories of her will always be long chats over a good question, rather quickly finding every other possible tangent. Incidentally, she made a very good cup of tea to mull it all over with. Margot taught me what it is to be intellectually curious and to explore the ideas that make you itchy. I felt that she was firmly of the belief that if something puzzled you, it was worth investigation. She supported me to write about wacky new reproductive technologies when others had implied it might not be worth the time. She encouraged me to think about a PhD, helping with the research proposal and writing references. I really don’t think I would have pursued the career I have or been where I am without her. What is remarkable, and a testament to her love for the subject and for her students, is quite how many people say the exact same.

